# Hypertension and Pneumonia: A Retrospective Analysis on the Length of Stay

**DOI:** 10.7759/cureus.110265

**Published:** 2026-06-04

**Authors:** Cassandra Blau, Diana Hamdan, Peter Reisdorf, Brandon Shin, Jack T Adcock, Nova Beyersdorfer, Kerry Johnson, John Paulson

**Affiliations:** 1 College of Osteopathic Medicine, Kansas City University of Medicine and Biosciences, Joplin, USA; 2 Mathematics, Missouri Southern State University, Joplin, USA

**Keywords:** comorbid, hospitalization, hypertension, length of stay, pneumonia

## Abstract

Background:* *Pneumonia is a serious respiratory infection that affects patients across all age groups. While it typically responds to treatment, patients with pre-existing comorbidities are at increased risk of poor outcomes. Hypertension (HTN) is a common condition that may coexist in patients with pneumonia. Although pneumonia and HTN have been studied separately, there is limited evidence regarding their combined effect on clinical outcomes, including hospital length of stay (LOS), treatment duration, and recovery trajectory.

Objectives: Identifying factors that influence the length of hospital stay in patients with pneumonia is essential for addressing the burden of hospitalization on both patients and healthcare systems. In this study, we compared the proportion of patients with an extended LOS (≥6 days) between patients with pneumonia with concomitant HTN and those without HTN.

Methods: A retrospective review of hospitalization data was conducted for 18,141 patients, who were divided into three groups: patients with both pneumonia and HTN (PXG), patients with pneumonia without HTN (P0G), and patients with HTN without pneumonia (0XG).

Results: The analysis revealed a statistically significant reduction in the proportion of patients with an extended LOS in the PXG cohort compared with the P0G cohort. Subgroup analysis indicated that this effect was driven by outcomes in patients aged >65 years (elderly).

Conclusions: Our findings suggest that hospitalization for patients with comorbid pneumonia and HTN is associated with a shorter LOS compared with patients with pneumonia without HTN. Unmeasured factors that may influence this association include the use of antihypertensive medications, adherence to lifestyle modifications, and a higher frequency of healthcare encounters.

## Introduction

Pneumonia is a leading cause of morbidity and mortality globally, particularly among vulnerable populations such as immunocompromised patients, those with multiple comorbidities, children, and older adults [1]. Hypertension (HTN) continues to increase in prevalence and remains the leading preventable risk factor for all-cause mortality worldwide. Antihypertensive medications are used to lower average global blood pressure levels [2]. Elevated blood pressure is a major risk factor for the development of several respiratory diseases; in particular, it increases the risk of pneumonia. In an epidemiological association study involving 107,310 patients with HTN, 9,969 developed pneumonia, and individuals with a genetic predisposition to a 5 mmHg increase in blood pressure had an increased risk of incident pneumonia [3]. The presence of HTN is also considered a predictor of cardiovascular events in patients with pneumonia [4]. Patients with both HTN and pneumonia are at greater risk of poor outcomes [5-8].

The literature suggests that antihypertensive medications, particularly angiotensin-converting enzyme inhibitors (ACEIs) and angiotensin receptor blockers (ARBs), may reduce the severity of pneumonia. ACEIs have been shown to lower the risk of pneumonia in elderly patients [9] and in those with intracerebral hemorrhage or cerebral infarction [10]. In a large cohort of patients with hypertension, the use of ACEIs and ARBs was associated with a lower risk of community-acquired pneumonia, with risk reduction varying according to duration of use [11]. In two other studies, ACEIs were superior to ARBs [12] and calcium channel blockers [13] in reducing the risk of pneumonia.

To further explore the association between pneumonia and HTN, length of stay (LOS) was reviewed and compared between patient groups. Patient outcomes are commonly evaluated using LOS as a clinical metric. A shorter LOS is often considered an indicator of an effective treatment plan, whereas an extended LOS (≥6 days) may suggest an insufficient treatment response or unforeseen complications.

This study highlights the relationship between HTN and pneumonia, focusing on LOS as a patient outcome measure. Understanding this association is crucial given the high prevalence of both conditions and their contribution to global morbidity and mortality. We found that comorbid essential HTN and pneumonia are associated with a lower proportion of patients with extended LOS. The objective of this study is to compare the proportion of patients with an extended LOS among patients with pneumonia with or without comorbid HTN.

## Materials and methods

An observational retrospective study evaluating the proportion of patients in each cohort with an extended LOS of six or more days was conducted at Freeman Health System (FHS) in rural Southwest Missouri between January 1, 2019, and December 31, 2022. All hospital discharges within this period were obtained from the FHS electronic medical records (EMR) system and de-identified for patients aged 18 years and older from hospitals in Joplin and Neosho, Missouri. Due to the retrospective nature of the study, informed consent was not required. Extracted EMR data were reviewed for completeness and consistency. Records with missing critical variables were excluded, while minor discrepancies were resolved through cross-referencing available fields when possible. No formal external validation dataset was used; however, internal checks were performed to ensure consistency in coding and categorization across the dataset. The reviewed EMRs were then sorted into appropriate cohorts of patients with pneumonia and those without pneumonia. Inclusion criteria were based on predefined International Classification of Diseases, Tenth Revision (ICD-10) codes [14] (Table 1).

**Table 1:** ICD-10 inclusion criteria for pneumonia and hypertension Source: [14].

ICD-10 codes	Description
J1000	Influenza due to other identified influenza virus with unspecified type of pneumonia
J1001	Influenza due to other identified influenza virus with the same other identified influenza virus pneumonia
J1008	Influenza due to other identified influenza virus with other specified pneumonia
J1100	Influenza due to unidentified influenza virus with unspecified type of pneumonia
J1108	Influenza due to unidentified influenza virus with specified pneumonia
J120	Adenoviral pneumonia
J121	Respiratory syncytial virus pneumonia
J122	Parainfluenza virus pneumonia
J123	Human metapneumovirus pneumonia
J1281	Pneumonia due to SARS-associated coronavirus
J1282	Pneumonia due to coronavirus disease 2019
J1289	Other viral pneumonia
J129	Viral pneumonia, unspecified
J13	Pneumonia due to *Streptococcus pneumoniae*
J14	Pneumonia due to *Haemophilus influenzae*
J150	Pneumonia due to *Klebsiella pneumoniae*
J151	Pneumonia due to *Pseudomonas*
J1520	Pneumonia due to *Staphylococcus*, unspecified
J15211	Pneumonia due to methicillin-susceptible *Staphylococcus aureus*
J15212	Pneumonia due to methicillin-resistant *Staphylococcus aureus*
J1529	Pneumonia due to other *Staphylococcus*
J153	Pneumonia due to *Streptococcus*, group B
J154	Pneumonia due to other streptococci
J155	Pneumonia due to *Escherichia coli*
J156	Pneumonia due to other Gram-negative bacteria
J157	Pneumonia due to *Mycoplasma pneumoniae*
J158	Pneumonia due to other specified bacteria
J159	Unspecified bacterial pneumonia
J160	Chlamydial pneumonia
J168	Pneumonia due to other specified infectious organisms
J17	Pneumonia in diseases classified elsewhere
J180	Bronchopneumonia, unspecified organism
J181	Lobar pneumonia, unspecified organism
J188	Other pneumonia, unspecified organism
J189	Pneumonia, unspecified organism
J84116	Cryptogenic organizing pneumonia
J851	Abscess of lung with pneumonia
J95851	Ventilator-associated pneumonia
I10	Essential (primary) hypertension

**Figure 1:**
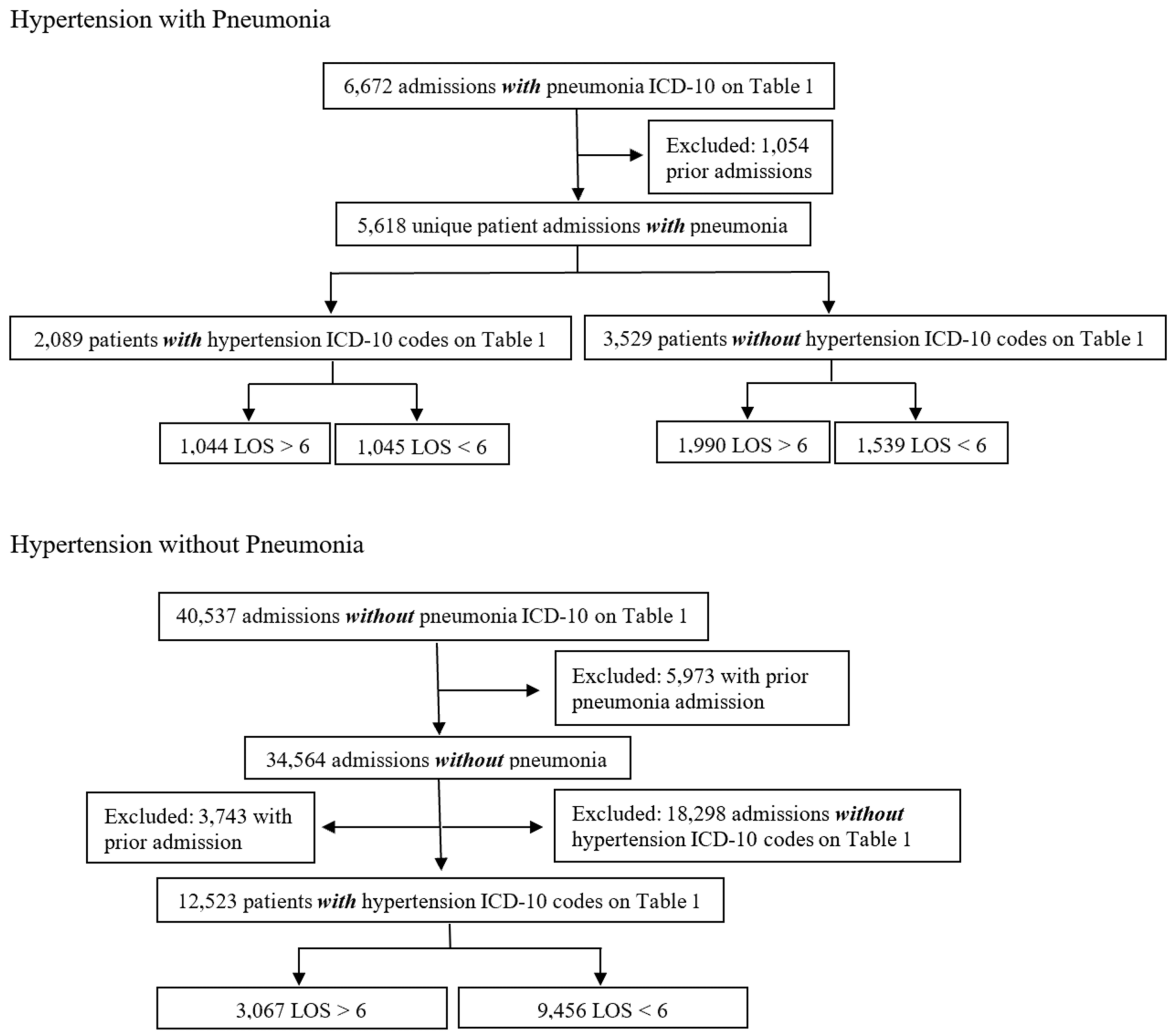
Flowchart of patient inclusion by cohort

Of the 6,672 (14.13%) admissions in the pneumonia cohort, 1,054 (2.44%) were excluded, resulting in 5,618 (11.90%) unique patient admissions (Figure 1). To address duplicate admissions, patients with multiple discharge records were identified, and a single admission was retained. The retained admission was the one with the longest LOS, as it was assumed to represent the most clinically significant episode. Patients were further stratified based on the presence of essential (primary) HTN (Table 1). Of the 40,537 (85.87%) admissions in the non-pneumonia cohort, 34,564 (73.21%) remained after excluding 5,973 (12.65%) records. Within this group, additional exclusions were applied: 3,743 (7.93%) prior admissions and 18,298 (38.76%) records without an HTN diagnosis. Ultimately, three cohorts were created for analysis using calculation formulas programmed in Excel (Microsoft Corp., Redmond, WA, USA): 2,089 (4.43%) patients with pneumonia and HTN (PXG), 3,529 (7.78%) patients with pneumonia without HTN (P0G), and 12,523 (26.53%) patients without pneumonia but with HTN (0XG). The cohorts were analyzed based on LOS of less than six days or six days or greater. Using Wald’s method, 95% confidence intervals (CIs) were calculated.

The total population analyzed consisted of 18,141 (38.43%) patients, of whom 51.29% (N = 9,304) were male and 48.71% (N = 8,837) were female. The P0G group had a mean age of 67.34 years, and patient-reported race was 92.21% (N = 3,254) White. The PXG group had a mean age of 67.12 years, and patient-reported race 94.02% (N = 1,964) White. Finally, the 0XG group had a mean age of 65.76 years, and patient-reported race was 94.74% (N = 11,864) White.

## Results

**Figure 2:**
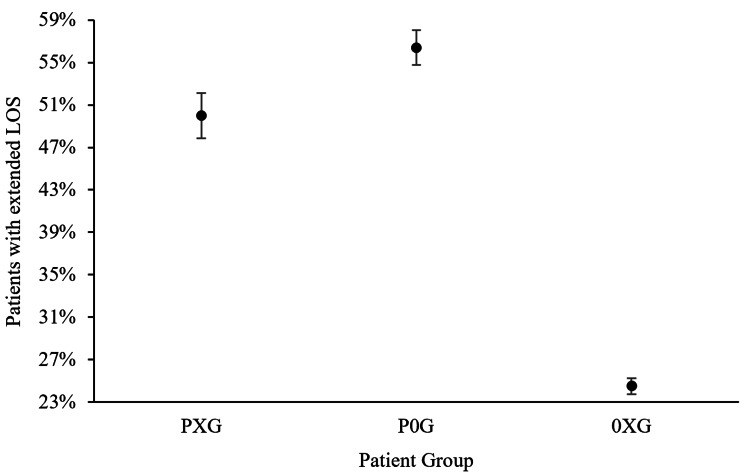
Percent of patients with extended hospital stays (LOS >6 days), with 95% CI of the patient population with pneumonia and HTN (PXG), with pneumonia without HTN (P0G), and without pneumonia with HTN (0XG)

**Figure 3:**
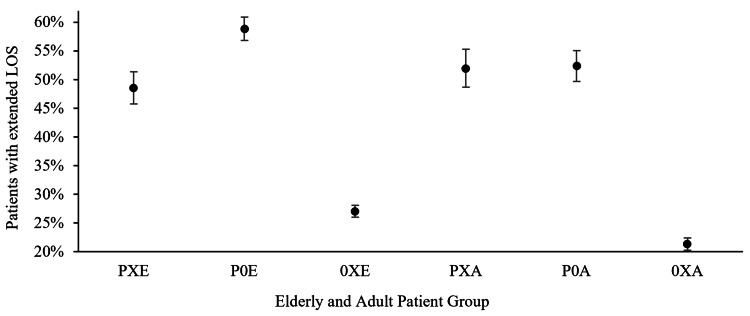
Percent of ≥65 years (elderly) (PXE, P0E, 0XE) and <65 years (adult) (PXA, P0A, 0XA) patient subgroups with extended length of hospital stay (LOS >6 days), with 95% CI of the patient population with pneumonia and HTN (PXE/PXA), with pneumonia without HTN (P0E/P0A), and without pneumonia with HTN (0XE/0XA)

The proportion of patients with an extended LOS (≥6 days) was estimated with 95% CIs: 54.75%-58.03% in the P0G group, 47.83%-52.12% in the PXG group, and 23.74%-25.24% in the 0XG group (Figure 2). When stratified by sex, the same pattern of CIs across groups was observed in both male and female subgroups. When stratified by age, subgroups were defined as ≥65 years (elderly; P0E and PXE) and <65 years (adult; P0A and PXA). In the elderly subgroup, the original pattern was maintained: the P0E group had a higher proportion of extended LOS than the PXE group, and this difference was statistically significant (p < 0.0001) (Figure 3). In contrast, the adult subgroup showed no significant difference between the PXA and P0A (p = 0.8529). A two-sample proportion hypothesis test demonstrated statistically significant differences across all group comparisons (p < 0.0001) (Table 2). Overall, the proportion of patients with extended LOS was 3.72%-9.11% lower in the PXG group compared with the P0G group.

**Table 2:** Two-sample proportion test group comparisons ^a^Group being compared: Sample 1 (S1) and Sample 2 (S2). ^b^Percent of patients in the group (S1 or S2) with extended length of stay (LOS >6 days). ^c^Numerical value reflecting percentages. ^d^95% CI for group comparison. This table shows the analysis between groups, comparing the number of patients in each group (PXG, P0G, 0XG) with extended hospital stays, with percent comparisons, 95% CIs, and the p-value reflecting the significance of the CI, utilizing a two-sample proportion summary hypothesis test.

Comparison (S1 vs S2)^a^	LOS sample 1^b^	LOS sample 2^b^	LOS sample 1 (N)^c^	LOS sample 2 (N)^c^	Sample 1 vs sample 2	Lower 95% CI for S1-S2^d^	Upper 95% CI for S1-S2^d^	p-value
PXG vs P0G	49.98%	56.39%	1044 of 2089	1990 of 3529	6.41%	3.72%	9.11%	<0.0001
PXG vs 0XG	49.98%	24.49%	1044 of 2089	3067 of 12523	25.49%	23.21%	27.76%	<0.0001
P0G vs 0XG	56.39%	24.49%	1990 of 3529	3067 of 12523	31.90%	30.10%	33.70%	<0.0001

## Discussion

This study investigated the impact of a comorbid diagnosis of HTN and pneumonia on LOS. The pneumonia with HTN group had a small yet statistically significant reduction in the percentage of the group with an extended LOS compared to the pneumonia without HTN group. Overall, the average age for the pneumonia cohort was 67.26 years, indicating that the pneumonia hospitalization burden is from elderly patients. Subgroup analysis by patient sex and age revealed that age but not sex was the primary driver for the trend. In the elderly group, an extended LOS was significantly lower in concomitant HTN and pneumonia patients compared with pneumonia without HTN patients. The adult subgroup yielded no significant difference in extended LOS between concomitant HTN and pneumonia compared with pneumonia without HTN. These results suggest that a comorbid diagnosis of HTN in a pneumonia patient was associated with a lower proportion of extended LOS.

Studies have previously identified sex, age, sepsis score, presence of comorbidities, and atypical presentation as potential predictors of LOS for pneumonia [15-19]. Our analysis presents a contradictory finding to previous research, suggesting that a comorbid diagnosis of HTN is associated with a reduced LOS for patients with pneumonia. This finding should be interpreted cautiously, given the potential for unmeasured confounding variables. Although based on prior studies, a possible explanation for our findings is the role of antihypertensive medications, such as ACEIs and ARBs, which have been associated with a reduced pneumonia hospitalization risk in case crossover studies [20]. Furthermore, long-term use of ACEI during pneumonia hospitalization was previously associated with shorter LOS [21].

Another hypothesized mechanism outside of medication is that the broader treatment regimen recommended for managing HTN may also play a role in improving patient outcomes during hospitalization. Treatment for HTN typically consists of a range of lifestyle changes to help modulate symptoms. These include frequent physical activity, healthy dietary habits, smoking cessation, limiting alcohol consumption, stress management, and improved sleep hygiene, all of which are utilized in HTN treatment guidelines [22]. As such, these lifestyle modifications may act synergistically with antihypertensive therapy to improve baseline health, thereby enhancing hospitalization outcomes [23]. Furthermore, a diagnosis of HTN may suggest prior encounters with healthcare providers. This patient experience may lead to improved patient disease awareness, education, lifestyle changes, and monitoring of overall health [24].

Pneumonia hospitalizations present a significant burden to patients and healthcare providers worldwide. Community-acquired pneumonia cases have higher admission rates and total costs compared with myocardial infarction and stroke hospitalizations in elderly populations [25]. This burden is complicated by the extended hospital stays often required for recovery. Reducing extended LOS lessens the risk of nosocomial infections and frees critical care bed capacity [26]. Identifying comorbidities and other factors that influence pneumonia-related LOS is essential for guiding interventions that optimize care.

This study is inherently limited by its retrospective nature and does not allow for random sampling. Furthermore, the data is obtained from rural Southwest Missouri and is representative of that population and possibly other rural midwestern areas. Otherwise, the study is limited in its generalizability to broader populations. The study also utilizes descriptive statistics, which limit the ability to conclude, make inferences, or determine causation. Several possible confounding variables were not accounted for in this analysis. This study did not provide evidence on pneumonia severity, specific antihypertensive use, patient comorbidity burden, or frailty. Further investigation could identify the role of these possible confounders on the outcomes seen in this study. There is sufficient evidence to warrant an in-depth investigation to further understand the array of potential comorbidities complicating pneumonia management. Understanding the interaction between comorbid pneumonia and HTN can reduce hospitalization burden and improve health outcomes among elderly populations.

## Conclusions

This study highlights a novel association between comorbid hypertension and a lower likelihood of extended LOS among older adults hospitalized with pneumonia. Prior literature has largely characterized comorbidities as predictors of poorer outcomes and prolonged hospitalization. These findings suggest that HTN is associated with a lower proportion of extended LOS in patients with pneumonia, although this observation may be influenced by unmeasured confounding variables. This underscores the need for further investigation into the interaction between chronic disease management and acute illness trajectories to guide strategies that reduce hospitalization burden and improve outcomes in patients with pneumonia.
